# Effects of OsteoKing on osteoporotic rabbits

**DOI:** 10.3892/mmr.2015.3551

**Published:** 2015-03-26

**Authors:** LIFEN DAI, HAIYING WU, SHAN YU, HONGBIN ZHAO, LANJIE XUE, MING XU, ZHIQIANG SHEN, MIN HU

**Affiliations:** 1 School of Pharmaceutical Science and Yunnan Key Laboratory of Pharmacology for Natural Products, Kunming Medical University, Kunming, Yunnan 650500, P.R. China; 2 Research Center for Molecular Medicine, Kunming University, Kunming, Yunnan 650214, P.R. China; 3 Department of Endocrinology, The Second Affiliated Hospital, Kunming Medical University, Kunming, Yunnan 650500, P.R. China; 4 Trauma Center of Emergency Medicine Department, First Affiliated Hospital of Kunming Medical University, Kunming, Yunnan 650032, P.R. China; 5 Faculty of Life Science and Technology, Kunming University of Science and Technology, Kunming, Yunnan 650500, P.R. China

**Keywords:** micro-computerized tomography, ovariectomy, bone mineral density, osteoporosis, Heng-Gu-Gu-Shang-Yu-He-Ji, OsteoKing

## Abstract

Heng-Gu-Gu-Shang-Yu-He-Ji, also known as OsteoKing, is used as a herbal Traditional Chinese Medicine for the treatment of bone disease, including femoral head necrosis and osteoarthritis. However, whether OsteoKing has anti-osteoporotic properties has remained to be elucidated. The purpose of the present study was therefore to investigate the effects of OsteoKing on ovariectomy-induced osteoporosis in rabbits. Female New Zealand white rabbits were randomly divided into an ovariectomized (OVX) group and a sham-surgery group. The rabbits in the OVX group were subjected to an ovariectomy, while the rabbits in the sham group were subjected to the removal of an area of fat near the two ovaries. Bone mineral density, mechanical properties, serum biochemical parameters and micro-architecture were examined at 150 days post-OVX to characterize the experimental animal model. Once the osteoporotic rabbit model had been established, the rabbits in the OVX group were divided into the following groups: Model group, nilestriol group and 300 and 600 mg/kg OsteoKing groups, containing 16 rabbits in each group. OsteoKing and nilestriol were administered orally. The bone mineral density, mechanical properties, serum biochemical parameters, histology and micro-architecture were examined using dual-energy X-ray absorptiometric analysis, mechanical assessments, enzyme-linked immunosorbent assays, histopathological evaluation and micro-computerized tomography examination following 60 days and 120 days of treatment, respectively. Treatment with OsteoKing led to an elevation in the bone mineral density of the vertebra and serum phosphorus levels, reduced serum concentrations of osteocalcin, procollagen type I N-terminal peptide, tartrate-resistant acid phosphatase 5b and cross-linked N-telopeptide of type I collagen, improved mechanical properties (maximum load, stiffness and energy absorption capacity), and micro-architecture of the lumbar vertebra in the OVX osteoporotic rabbit model following treatment for 120 days. In conclusion, it was demonstrated that OsteoKing is effective in the prevention of estrogen deficiency-associated bone loss and may be a promising drug for the treatment of post-menopausal osteoporosis.

## Introduction

Osteoporosis is a chronic, metabolic and systemic skeletal disease characterized by low bone mineral density (BMD) and micro-architectural deterioration, resulting in increased bone fragility and fracture risk (1,2). It is estimated that >200 million individuals worldwide suffer from osteoporosis and the prevalence is continuing to increase with the growing elderly population (3). The incidence of osteoporosis is 2–4 times higher in females than that in males due to a sharp decrease in ovarian estrogen production, which causes rapid bone loss during the first decade following the menopause (4). Bone fracture, which is the most serious consequence of osteoporosis, is associated with high economic costs and substantial morbidity and mortality; therefore, the prevention and treatment of this condition are of great importance (5). Current drug treatments for the prevention and treatment of post-menopausal osteoporosis include estrogen, selective estrogen receptor modulators, calcitonin and bisphosphonates (4,6,7). Although these agents are effective in preventing bone loss, they are not the ideal treatments due to their adverse side effects on the breast and the gastrointestinal and cardiovascular systems, as well as increasing the risk of endometrial or ovarian cancer (4,8–12). Novel drugs based on medicinal herbs and natural products, and which possess fewer side effects, are urgently required (13). Traditional Chinese Medicines have been widely used in the prevention and treatment of post-menopausal osteoporosis, and as these medicines are prepared from medicinal plants, are a source of numerous bioactive compounds and are preferred by patients, they are more suitable for long-term use compared with chemically synthesized medicines (14).

Heng-Gu-Gu-Shang-Yu-He-Ji (OsteoKing) is a formulation composed of numerous types of medicinal herbs (Pericarpium Citri reticulatae, Carthamus tinctorius L., Radix notoginseng, Eucommia ulmoides Oliv., Radix ginseng, Radix Astragali Mongolici and Carapax trionycis) based on a concoction originating from Yunnan Province in China and has been used for >100 years. It has a notable effect in the treatment of bone diseases, particularly for femoral head necrosis, prolapse of the lumbar intervertebral disc and osteoarthritis, and was approved by the Chinese State Food and Drug Administration in 2002 (15,16). Previous studies by our group demonstrated that OsteoKing is able to elevate the gene expression of core binding factor α 1 and vascular endothelial growth factor, and improve the micro-architecture in the necrotic femoral head of rabbits (17–20). Clinical studies have demonstrated that OsteoKing has an effect in preventing fracture and treating ischemic necrosis of the femoral head in humans (21–23). However, no studies have been performed thus far to investigate whether OsteoKing has any anti-osteoporotic activity. The present study was conducted to investigate the effects of OsteoKing on an osteoporosis model of ovariectomized (OVX) rabbits.

## Materials and methods

### Drugs and reagents

The OsteoKing concoction was prepared according to the Chinese Pharmacopeia (China Pharmacopeia Committee, 2002) and was supplied by Crystal Natural Pharmaceutical Co. (Kunming, China). Briefly, *Pericarpium Citri reticulatae* (10 g), *Carthamus tinctorius* L. (15 g), *Radix Notoginseng* (30 g), *Eucommia ulmoides* Oliv. (30 g), *Radix Ginseng* (20 g), *Radix Astragali mongolici* (40 g) and *Carapax Trionycis* (10 g) were ground into a coarse powder and immersed in 10X (10 l/kg) distilled water for 12 h at room temperature, and then boiled using a distillation apparatus for 1 h. This process was repeated twice and for the second and third extraction, the residue from the previous extraction was filtered and the same extraction procedures were applied. Thereafter, the combined extracts were filtrated and evaporated using a rotary evaporator at 50°C to a relative density of 1.03–1.04 g/cm^3^, centrifuged for 30 min at 1,450 × g and the supernatant obtained was centrifuged once again after standing for 12 h. Subsequently, the pH was adjusted to 4.0–6.0 using NaOH (Shaihai Experiment Co., Shanghai, China), distilled water was added to a total volume of 1,000 ml and the product was filtrated prior to usage. Nilestriol was purchased from Shanghai New Hualian Pharmaceutical Co. Ltd. (Shanghai, China). Rabbit enzyme-linked immunosorbent assay (ELISA) kits for measurement of the serum concentrations of osteocalcin (OC), procollagen type I N-terminal peptide (PINP), tartrate-resistant acid phosphatase 5b (TRAP5b), cross linked N-telopeptide of type I collagen (NTX) with a sensitivity of 0.3 ng/ml, 0.2 ng/ml, 19.5 *μ*U/ml and 0.78 pmol/ml, respectively, were purchased from Wuhan Huamei Bioengineering Co. (Wuhan, China), and the intra-assay and inter-assay coefficients of variability of all the ELISA kits were <8 and 10%, respectively.

### Animals

A total of 101 female New Zealand white rabbits aged 6 months were obtained from the Animal Center of Kunming Medical University (Kunming, China). Their body weight ranged between 2.5 and 3.0 kg. The animals were housed at a constant temperature (20–25°C), humidity (40–70%) and light-dark cycle (12/12 h). Tap water was available *ad libitum*, while standard rabbit chow was restricted to 50 g per day. All experiments were conducted under the National Institutes of Health Guide for the Care and Use of Laboratory Animals and approved by the Ethics Committee (Animal Care and Use Committee) of Kunming Medical University. All efforts were made to minimize the pain and suffering of the animals.

### Experimental protocol

Following a two-week acclimation period, the animals were randomly allocated into an OVX model group (OVX group, n=76) and a sham-surgery group (sham group, n=25). Animals of the OVX group underwent a bilateral ovariectomy as previously described under general anesthesia with an intravenous injection of sodium pentobarbital (30 mg/kg; Shanghai Westang Bio-tech Co., Ltd., Shanghai, China), while the sham surgery group was subjected to a procedure involving exposure of the ovaries without excision (24). Post-operatively, all animals (Animal Center of Kunming Medical University, Kunming, China) fasted for 12 h and sodium benzylpenicillin (0.3 million IU/kg; Wuhan Dahua Pharmaceutical Co., Ltd., Wuhan, China) was administered via an intramuscular injection for five days to prevent infection (25). However, one rabbit from the sham surgery group and two rabbits from the OVX group died within 150 days following surgery. To characterize the experimental animal model, six animals from each group were selected randomly 150 days following OVX to determine the BMD of their vertebrae, serum biochemical parameters, mechanical properties and micro-architecture of the lumbar vertebra. Once the osteoporotic rabbit model was established, 16 rabbits from the sham group were randomly selected to continue with the study, and 64 rabbits from the OVX group were randomly divided into four groups: Model group (Model), OVX with nilestriol group (nilestriol), OVX with 300 mg/kg OsteoKing group (OsteoKing 300) and OVX with 600 mg/kg OsteoKing (OsteoKing 600) group, containing 16 rabbits each. OsteoKing was administered orally once every other day with the dose at 300 mg/kg approximating the clinical application dose for humans (19,20). Nilestriol (Shanghai Hualian Pharmaceutical Co., Ltd., Shanghai, China) was administered orally at a dose of 0.5 mg/kg once weekly (26,27). Rabbits of the sham group and model group were treated with deionized water. All animals were weighed and the doses were adjusted weekly. At 60 days and 120 days after treatment, six randomly selected rabbits from each group were sacrificed and the effects of OsteoKing or nilestriol on the BMD of the vertebrae, serum biochemical parameters and mechanical properties, histology, and micro-architecture of the lumbar vertebra were recorded.

### BMD analysis

The rabbits were anesthetized with an intravenous injection of sodium pentobarbital (30 mg/kg) and the BMD of the vertebrae was measured *in vivo* using dual-energy X-ray absorptiometry (DXA; Lunar Prodigy Advance; GE Lunar, Madison, WI, USA) as described previously (22) Specific software for small animals (GE Medical Systems, enCORE 2004 software; cersion 8.80.001) was used. BMD measurements were performed following 150 days bilateral ovariectomy (0 days treatment) and 60, 120 days treatment, respectively.

### Mechanical assessment

The second lumbar vertebra was harvested at days 0, 60 and 120 of treatment, frozen at −20°C prior to the assay and the mechanical properties were measured as described previously (25,28). The bones were thawed at room temperature prior to the mechanical assessments and moisture levels were retained with the use of a moist gauze soaked in 0.9% NaCl solution (The Third Chemical Reagent Factory, Tianjin, China) throughout the entire assessment period. The vertebrae were prepared by cutting off the end plates from the vertebral body to create parallel planar surfaces using a diamond wafer saw (VT1200, Leica Microsystems GmbH, Wetzlar,, Germany). The vertebral samples were then placed centrally between two parallel steel plates attached to a materials-testing machine (Instron System 5565; Instron, Norwood, MA, USA) and assessed along the longitudinal axis at a constant compressive speed of 1 mm/min (25,29). The specimens were loaded until the specimen succumbed to the strain/weight and the mechanical parameters (maximum load, displacement, stiffness and energy absorption capacity) were calculated from the load-displacement curves. Briefly, the maximum load (N) was considered as the maximum force on the curve; furthermore, displacement (mm) and the ultimate deformity prior to failure and stiffness (N/mm) were determined from the slope of the linear portion, and the area under the load-displacement curve was defined as the energy absorption capacity (mJ) (28,30).

### Biochemical analysis of serum

Blood samples were collected from the central ear artery 150 days following bilateral ovariectomy (day 0 of treatment) and at days 60 and 120 of treatment, respectively, after an overnight fast, consistently between 09:00 and 11:00 A.M. The serum was promptly separated and stored at −80°C prior to the assay (31). The serum calcium (Ca^2+^) and inorganic phosphorus (P) levels were determined using a biochemical automatic analyzer (Hitachi 7080; Hitachi Ltd., Tokyo, Japan), and the serum concentrations of OC, PINP, TRAP5b and NTX were measured using rabbit ELISA kits. All samples were run in the same assay unless an individual value required repeating.

### Histopathological evaluation

The sections of the third lumbar vertebrae, which were harvested 60 or 120 days following treatment, were prepared as described previously (32,33). Briefly, samples were fixed in 10% neutral formal-saline (Day Ning Chemical Reagent Co., Ltd., Jining, Shandong, China) for five days, dehydrated in a graded ethanol series, and embedded in paraffin following decalcification in 10% ethylene diamine tetraacetic acid (Kunming Pegatron Yang Technology Co., Ltd., Kunming, China) for 30 days. Subsequently, the blocks were cut into 5-*μ*m slices perpendicular to the longitudinal axis at the middle of the lumbar vertebra. The morphology of the sections was examined under a light microscope (Nikon AZ100; Nikon, Tokyo, Japan) following staining with hematoxylin and eosin (Wuhan Baihao Biological Technology Co., Ltd., Wuhan,China).

### Micro-computerized tomography (MicroCT) examination

The first lumbar vertebra, which was harvested 150 days after the bilateral ovariectomy (day 0 of treatment) and 60 days or 120 days after treatment was cleaned of adherent soft tissues and preserved in sealed plastic bags at −20°C prior to the assay (34). The MicroCT examination of the first lumbar vertebra was performed using a MicroCT system (*μ* CT 80, SCANCO Medical, Brüttisellen, Switzerland) as previously described (34,35), and the analytical conditions were 55 kV with 72 *μ*A leakage. The lumbar vertebra was scanned and ~500 transverse consecutive sections of 35-*μ*m thickness were obtained from each lumbar vertebra using a 2048×2048 matrix. The volume of interest was selected as a region 100 slices subsequent to 50 slices away from the cranial endplate (36). Within these slices, the region of the vertebral body, excluding the cortical bone by the boundaries defined by the endocortical bone surfaces, was selected and constructed three-dimensionally. Following setting the same threshold, the structural parameters, including bone volume/total volume (BV/TV), bone surface/bone volume (BS/BV), trabecular thickness (Tb. Th), trabecular separation (Tb.Sp) and trabecular number (Tb.N) were measured automatically for each specimen using the plate-model data with the SCANCO microtomographic software package version 6.0 (SCANCO Medical) (35).

### Statistical analysis

All experimental data were assessed using the statistical system SPSS 17.0 (SPSS, Inc., Chicago, IL, USA) and values are expressed as the mean ± standard deviation. Differences in the mean values of BMD, serum biochemical parameters, mechanical parameters and structural parameters between the two groups 150 days after OVX were compared using an independent-samples t-test, and those between five groups at the same time -point after treatment were performed using one-way analysis of variance with the Bonferroni post hoc test. P<0.05 was considered to indicate a statistically significant difference.

## Results

### BMD measurements

The effects of OsteoKing or nilestriol on the BMD of the vertebrae are presented in Table I. The BMD of the vertebra in the OVX group 150 days after the surgery decreased by 14.0% (P<0.01) compared with that in the sham group. No significant differences were observed in the BMD between any treatment group and the model group 60 days after treatment (P>0.05). At 120 days of treatment, the BMD in the group subjected to OVX and treated with 600 mg/kg OsteoKing was significantly higher than that in the model group (P<0.01), almost identical to that in the sham group and similar to that in the OVX with nilestriol group, while the improvement of BMD in the group subjected to OVX and treated with 300 mg/kg OsteoKing was not significant (P>0.05).

### Mechanical properties of the lumbar vertebrae

The results of the vertebral compression assessment are shown in Table II. The values of maximum load, displacement, stiffness and energy in the OVX group 150 days after surgery decreased by 44.7, 6.8, 44.3 and 50.3%, respectively, compared with those in the sham group (P<0.01, P<0.05, P<0.01 and P<0.01, respectively). At 60 days following treatment, the values of maximum load and stiffness were significantly higher in the group subjected to OVX and treated with 300 mg/kg OsteoKing than those in the model group (P<0.05), but remained significantly lower than those in the sham group (P<0.05). The values of maximum load and stiffness were also significantly higher in the group subjected to OVX and treated with 600 mg/kg OsteoKing than those in the model group (P<0.01), and no significant difference was identified (P>0.05) compared with the sham group with the exception of the energy levels (P<0.05). Following 120 days of treatment, the values of maximum load, stiffness and energy were significantly higher in the OsteoKing-treated group than those in the model group (P<0.01 or P<0.05), and no difference was identified compared with those in the sham group (P>0.05). Similar gradual increases in maximum load, stiffness and energy were observed in nilestriol-treated group. No significant difference was observed in the value of displacement between any groups at any time-point of treatment (P>0.05).

### Serum biochemical parameters

The results of the serum biochemical assessment are shown in Table III. The levels of OC (+37.6%), PINP (+56.9%), TRAP5b (+45.2%) and NTX (+40.0%) were significantly higher (P<0.01) and the levels of serum Ca^2+^ (P<0.05) and P (P<0.01) were markedly lower in the model group than those in the sham group 150 days after the surgery, indicating the induction of a high bone turnover following OVX. No significant difference was identified in the serum Ca^2+^ levels among the groups at the same time-points. Following 60 days of treatment, the levels of OC, PINP, TRAP5b and NTX decreased by 8.6, 8.3, 10.0 and 16.2%, respectively, in the group subjected to OVX and treated with 300 mg/kg OsteoKing, and decreased by 16.4, 20.6, 18.7 and 22.2%, respectively, in the group subjected to OVX and treated with 600 mg/kg OsteoKing as compared with those in the model group at 150 days after OVX; however, the decrease in the levels of all bone turnover biomarkers in the OsteoKing-treated groups was not significantly different compared with those in the model group at the same time-point (P>0.05). The levels of serum P in the group subjected to OVX and treated with 600 mg/kg OsteoKing were significantly higher (P<0.05) than those in the model group. At 120 days following treatment, compared with those in the model group at the same time-point, the levels of OC, PINP, TRAP5b and NTX in the OsteoKing-treated group decreased significantly and the levels of serum P increased significantly, almost recovering to the normal levels. Nilestriol treatment had a similar effect to the two doses of OsteoKing in reducing bone turnover and increasing serum P levels.

### Histological analysis of lumbar vertebrae

**Figure 1:**
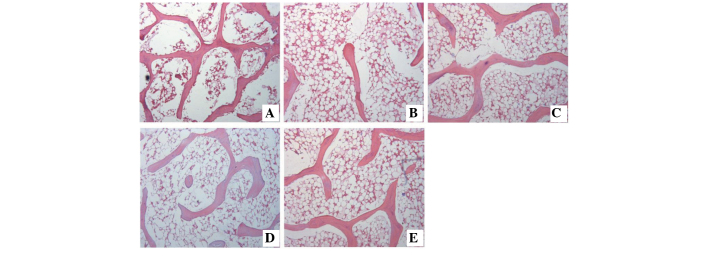
Photomicrograph of the third lumbar vertebra following 60 days of treatment (hematoxylin and eosin stain; magnification, ×40). (A) Sham group; (B) Model group; (C) OVX with nilestriol group; (D) OVX with 300 mg/kg OsteoKing group; (E) OVX with 600 mg/kg OsteoKing group. OVX, ovariectomized.

**Figure 2:**
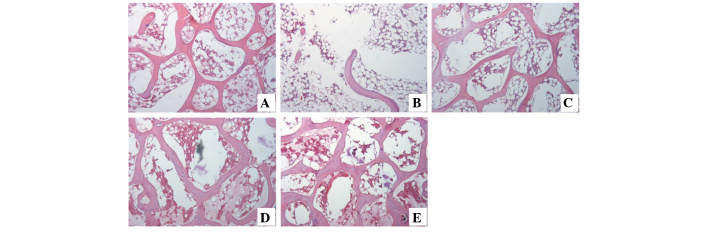
Photomicrograph of the third lumbar vertebra following 120 days of treatment (hematoxylin and eosin stain; magnification, ×40). (A) Sham group; (B) Model group; (C) OVX with nilestriol group; (D) OVX with 300 mg/kg OsteoKing group; (E) OVX with 600 mg/kg OsteoKing group. OVX, ovariectomized.

Under the light microscope, following 60 and 120 days of treatment, the histology of the third lumbar vertebra of the sham group exhibited the normal size, shape, density and architecture of the trabecular bone (Figs. 1A and 2A), while sections of the OVX group exhibited sparse, disrupted, spacing-enlarged and area-diminished trabecular bone tissue (Figs. 1B and 2B). The OsteoKing-treated group exhibited partial trabecular restoration following 60 days of treatment (Fig. 1D and E) and exhibited almost complete restoration of normal architecture following 120 days of treatment (Fig. 2D and E); similar effects were also observed in the nilestriol treatment group (Figs. 1C and 2C).

### MicroCT evaluation

**Figure 3:**
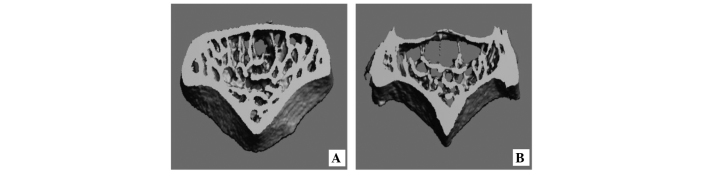
Three-dimensional reconstruction using micro-computerized tomography of the first lumbar vertebra 150 days after OVX. (A) Sham group; (B) OVX group. OVX, ovariectomized.

**Figure 4:**
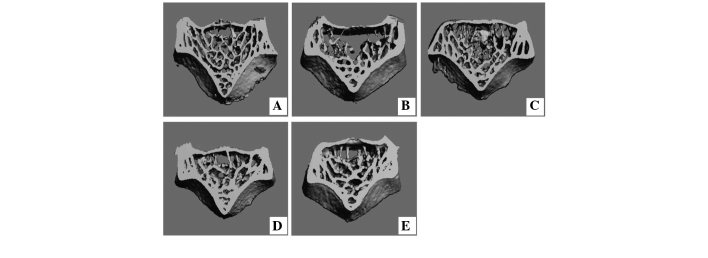
Three-dimensional reconstruction using micro-computerized tomography of the first lumbar vertebra following 60 days of treatment. (A) Sham group; (B) Model group; (C) OVX with nilestriol group; (D) OVX with 300 mg/kg OsteoKing group; (E) OVX with 600 mg/kg OsteoKing group. OVX, ovariectomized.

**Figure 5:**
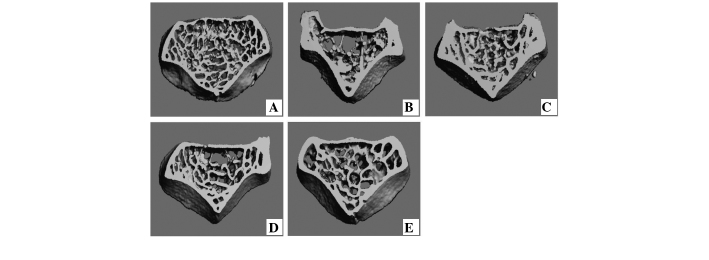
Three-dimensional reconstruction using micro-computerized tomography of the first lumbar vertebra following 120 days of treatment. (A) Sham group; (B) Model group; (C) OVX with nilestriol group; (D) OVX with 300 mg/kg OsteoKing group; (E) OVX with 600 mg/kg OsteoKing group. OVX, ovariectomized.

At 150 days after ovariectomy, the rabbits of the OVX group exhibited lower values for BV/TV, Tb.Th and Tb.N, and higher values for BS/BV and Tb.Sp (P<0.01), when compared with those in the sham group (Table IV and Fig 4). Treating OVX rabbits with OsteoKing (300 or 600 mg/kg) or nilestriol partly abrogated the OVX-mediated changes in the abovementioned parameters, resulting in levels similar to those in the sham group (Table IV). Typical three-dimensional reconstructed MicroCT images of the first lumbar vertebra (Figs. 3, 4 and 5), including the cortical bone, revealed differences in trabecular micro-architecture among the various groups. Images of the representative samples with the BV/TV closest to the mean BV/TV were reconstructed in each group (32).

## Discussion

OsteoKing is a Traditional Chinese Medicine, which is widely used in the treatment of bone disease, particularly for femoral head necrosis, prolapse of the lumbar intervertebral disc and osteoarthritis. Previous studies have demonstrated that OsteoKing has an effect on the prevention of fracture and the improvement of the micro-architecture in the necrotic femoral head of rabbits, which indicates that OsteoKing may have anti-osteoporotic effects (17,18,21,23). The present study was the first, to the best of our knowledge, to demonstrate the beneficial effects of OsteoKing against the reduction of bone mass and bone strength, and the deterioration of the micro-architecture of the bone induced by ovariectomy in rabbits.

Experimental animal models are important in improving knowledge of the aetiology, pathophysiology and diagnosis of osteoporosis, as well as in the prevention of the condition and development of therapeutics (2). It is well known that estrogen deficiency is an important risk factor in the pathogenesis of osteoporosis and estrogenic deprivation has been the most commonly used experimental model of osteoporosis in animals (33,37). Although the ovariectomy rat model is the most frequently used animal model of osteoporosis, rats do not experience a natural menopause, fail to achieve true skeletal maturity, lack the Haversian system and remodeling differs from that in humans (38–41). By contrast, rabbits have a short developmental period and fast bone turnover, achieve skeletal maturity shortly after reaching complete sexual development at ~six months of age, and exhibit an active Haversian remodeling; therefore, rabbits are often selected for the investigation of osteoporosis (25,37,41). Osteoporotic rabbit models induced by OVX or glucocorticoid (GC) alone and OVX combined with GC have been used to investigate the effects of loss of bone mass and have exhibited promising results of reductions of BMD (24,25,37,42,43). In the present study, six-month-old rabbits underwent bilateral ovariectomy alone (without GC administration), and after 150 days, the BMD of the vertebrae in the OVX group decreased by 14.0%, which was significantly lower compared with that in the sham group (P<0.01). Furthermore, a significant reduction of mechanical strength in the vertebral load compression assessments and micro-architectural deterioration of the lumbar vertebra were observed in rabbits of the OVX group. The osteoporotic rabbit model induced by OVX was successfully established after a longer period than that reported in previous studies (24,25,28,37,38).

Bone turnover markers reflect the rates of bone resorption and bone formation in the whole body, and they provide a representative index of the overall skeletal bone loss (44–47). Estrogen deficiency following a natural or artificial menopause results in an increase in the levels of markers of bone remod-eling and bone turnover (48). Previous studies on menopausal women demonstrated that the increase in the markers of bone resorption (of 50–150%) is rapid and precedes the increase (of 50–100%) in markers of bone formation by several months. This imbalance and disproportionately high rate of bone resorption compared with formation exists within several years following ovariectomy and remains in late postmenopausal women (48–50). Similar changes were observed in the present study: 150 days after ovariectomy, the expression of OC and PINP, which are the biomarkers of bone formation, increased by 37.6 and 56.9% respectively, and TRAP5b and NTX, which are biomarkers of bone resorption, increased by 45.2 and 40.0%, respectively, as compared with those in the sham group. Following treatment with OsteoKing, the levels of all the above bone turnover biomarkers returned to a normal level and the rate of decrease in bone resorption markers was slightly faster, which indicated that OsteoKing may prevent bone loss by suppressing bone resorption. However, long-term suppression of bone turnover may eventually lead to an accumulation of fatigue-induced damage, as was observed following bisphosphonate treatment (51,52). Therefore, although the levels of bone turnover biomarkers returned to normal levels, it was important to measure the mechanical properties and micro-architecture of bone.

Compression testing of the lumbar vertebrae is recommended for the assessment of the mechanical properties of cancellous bone (28). The biomechanical definition of bone fragility includes at least three components: Strength (maximum load), brittleness (reciprocal of the displacement) and work to failure (energy absorption) (25,52). A fourth biomechanical measure, stiffness, is also used to assess the mechanical integrity of bone, but is not a direct measure of fragility (52). In the present study, the strength, brittleness, stiffness and energy absorption of the lumbar vertebrae decreased significantly in the OVX group compared with that in the sham group 150 days after ovariectomy. Following OsteoKing treatment, the strength, stiffness and energy absorption increased gradually and almost recovered to normal levels, but no effect on the brittleness was observed. This was consistent with a previous study, which demonstrated that it was rare for a treatment to improve strength and decrease brittleness simultaneously (53).

Previous studies have demonstrated that BMD and the aspects of the trabecular micro-architecture affect trabecular bone strength (53). In the present study, the BMD of the vertebrae decreased significantly and deteriorated micro-architecture was observed in the MicroCT analysis of the lumbar vertebrae 150 days after OVX. Increased vertebral BMD and trabecular restoration of the lumbar vertebrae were observed following OsteoKing treatment by the DXA and histopathological evaluation, respectively. Furthermore, MicroCT analysis demonstrated an increased travbecular BV/TV, Tb.Th, Tb.N and decreased BS/BV, Tb.Sp following treatment with OsteoKing compared with those in the model group. These results were consistent with the consequences of the mechanical assessment and previous studies, which indicated that the suppression of bone turnover did not lead to bone damage (52).

In conclusion, OsteoKing was able to elevate the BMD of the vertebrae, reduce the bone turnover rate, restore the trabecular network and ameliorate the mechanical properties of lumbar vertebra in a rabbit model of ovariectomy-induced osteoporosis. These findings suggested that OsteoKing is potentially useful in the treatment of postmenopausal osteoporosis, which occurs in women as a result of estrogen deficiency.

## Figures and Tables

**Table I:** Effect of OsteoKing or nilestriol on bone mineral density (g/cm^2^) of vertebrae in ovariectomized rabbits.

Time-point (days)	Sham group	Ovariectomized group			
		Model	nilestriol	OsteoKing 300	OsteoKing 600
0	0.265±0.016		0.228±0.017^a^		
60	0.264±0.026	0.225±0.014^a^	0.245±0.011	0.238±0.011	0.248±0.017
120	0.262±0.021	0.227±0.015^a^	0.262±0.011^b^	0.249±0.011	0.266±0.018^b^

Values are expressed as the mean ± standard deviation (n=6).

a P<0.01 vs. sham group;

b P<0.01 vs. model group. OsteoKing 300/600, ovariectomized and treated with 300/600 mg/kg OsteoKing every other day.

**Table II:** Effect of OsteoKing or nilestriol on biomechanical parameters of the second lumbar vertebra in ovariectomized rabbits.

Time-point (days)	Group	Maximum load (N)	Displacement (mm)	Stiffness (N/mm)	Energy (mJ)
0	Sham	615.8±61.7	0.676±0.013	1504.0±125.3	208.4±42.5
	OVX	340.6±67.6^b^	0.630±0.035^a^	837.7±229.1^b^	103.5±24.5^b^
60	Sham	611.6±64.6	0.678±0.015	1498.5±97.6	201.1±39.1
	Model	336.5±64.6^b^	0.615±0.047	826.3±220.6^b^	96.5±23.9^b^
	Nilestriol	499.4±61.7^d^	0.618±0.036	1259.1±173.4^d^	135.6±25.8^b^
	OsteoKing 300	474.2±69.1^a^,^c^	0.621±0.041	1153.9±148.3^a^,^c^	127.3±19.2^b^
	OsteoKing 600	515.5±65.9^d^	0.639±0.051	1267.2±152.6^d^	144.5±26.3^a^
120	Sham	615.3±44.7	0.670±0.017	1489.1±79.2	197.2±35.7
	Model	331.8±61.9^b^	0.612±0.043	819.0±221.8^b^	92.8±23.5^b^
	Nilestriol	573.9±46.6^c^	0.624±0.031	1422.8±104.4^d^	164.3±28.4^d^
	OsteoKing 300	548.3±60.4^c^	0.625±0.058	1406.2±120.4^d^	158.5±30.5^a^
	OsteoKing 600	589.3±55.0^c^	0.634±0.052	1467.1±102.1^d^	170.7±42.4^d^

Values are expressed as the mean ± standard deviation (n=6).

a P<0.05,

b P<0.01 vs. sham group;

c P<0.05,

d P<0.01 vs. model group. OsteoKing 300/600, ovariectomized and treated with 300/600 mg/kg OsteoKing every other day.

**Table III:** Effect of OsteoKing or nilestriol on serum biochemical parameters in OVX rabbits.

Time-point (days)	Group	Ca^2+^ (mmol/l)	P (mmol/l)	OC (ng/ml)	PINP (ng/ml)	TRAP5b (mU/ml)	NTX (pmol/ml)
0	Sham	3.43±0.07	1.59±0.10	7.47±1.19	2.16±0.52	5.98±0.85	7.62±0.92
	OVX	3.29±0.12^a^	1.26±0.13^b^	10.28±1.12^b^	3.39±0.71^b^	8.68±1.17^b^	10.67±1.05^b^
60	Sham	3.41±0.10	1.59±0.13	7.53±0.93	2.11±0.60	6.01±1.29	7.50±0.91
	Model	3.34±0.14	1.28±0.15^b^	10.05±1.45^a^	3.36±0.60^a^	8.59±0.92^b^	10.45±1.51^b^
	Nilestriol	3.38±0.10	1.57±0.12^c^	8.41±1.46	2.72±0.80	6.89±0.95	8.18±1.34
	OsteoKing 300	3.36±0.16	1.48±0.18	9.40±1.52	3.11±0.49	7.81±1.06	8.94±1.44
	OsteoKing 600	3.37±0.14	1.56±0.12^c^	8.59±1.38	2.69±0.73	7.06±1.15	8.30±1.26
120	Sham	3.41±0.10	1.58±0.11	7.45±0.90	2.06±0.56	5.81±0.95	7.54±0.90
	Model	3.33±0.08	1.29±0.09^b^	9.79±1.22^b^	3.26±0.77^a^	8.30±1.27^b^	10.26±1.25^b^
	Nilestriol	3.41±0.10	1.58±0.09^d^	7.63±0.83^c^	1.88±0.46^c^	5.67±0.61^c^	7.56±0.82^d^
	OsteoKing 300	3.39±0.09	1.58±0.10^d^	8.35±0.93	2.22±0.84	6.26±0.89^c^	8.22±1.05^c^
	OsteoKing 600	3.40±0.14	1.59±0.08^d^	7.75±1.16^c^	2.05±0.63^c^	5.58±0.77^c^	7.55±1.47^d^

Values are expressed as the mean ± standard deviation (n=6).

a P<0.05,

b P<0.01 vs. sham group;

c P<0.05,

d P<0.01 vs. model group. OsteoKing 300/600, ovariectomized and treated with 300/600 mg/kg OsteoKing every other day. OVX, ovariectomized; OC, osteocalcin; PINP, procollagen type I N-terminal peptide; TRAP5b, tartrate-resistant acid phosphatase 5b; NTX, cross-linked N-telopeptide of type I collagen.

**Table IV:** Effect of OsteoKing or nilestriol on the micro-architecture parameters of the first lumbar vertebra in ovariectomized rabbits.

Time-point (days)	Group	BV/TV (%)	BS/BV (1/mm)	Tb.Th (mm)	Tb.Sp (mm)	Tb.N (1/mm)
0	Sham	0.385±0.052	6.161±0.312	0.325±0.017	0.891±0.053	1.178±0.102
	Ovariectomized	0.193±0.048^b^	7.489±0.454^b^	0.268±0.016^b^	1.278±0.075^b^	0.713±0.130^b^
60	Sham	0.386±0.047	6.269±0.298	0.320±0.015	0.866±0.080	1.203±0.093
	Model	0.200±0.043^b^	7.405±0.334^b^	0.271±0.012^b^	1.244±0.093^b^	0.735±0.129^b^
	Nilestriol	0.291±0.049^a^,^c^	6.784±0.457	0.296±0.020	1.018±0.099^d^	0.977±0.102^a^,^c^
	OsteoKing 300	0.268±0.046^b^	6.928±0.411	0.290±0.017	1.070±0.123^a^	0.922±0.118^b^
	OsteoKing 600	0.295±0.046^b^	6.668±0.411^c^	0.301±0.019^c^	1.038±0.096^c^	0.975±0.099^a^,^d^
120	Sham	0.387±0.042	6.173±0.265	0.324±0.014	0.864±0.081	1.190±0.098
	Model	0.198±0.041^b^	7.512±0.368^b^	0.267±0.013^b^	1.254±0.089^b^	0.737±1.122^b^
	Nilestriol	0.348±0.051^d^	6.319±0.283^d^	0.317±0.014^d^	0.862±0.111^d^	1.096±0.124^d^
	OsteoKing 300	0.314±0.053^d^	6.623±0.440^d^	0.303±0.020^d^	0.906±0.106^d^	1.034±0.126^d^
	OsteoKing 600	0.345±0.060^d^	6.383±0.356^d^	0.314±0.018^d^	0.879±0.098^d^	1.094±0.132^d^

Values are expressed as the mean ± standard deviation (n=6).

a P<0.05,

b P<0.01 vs. sham group;

c P<0.05,

d P<0.01 vs. model group. OsteoKing 300/600, ovariectomized and treated with 300/600 mg/kg OsteoKing every other day. BV/TV, bone volume/total volume; BS/BV, bone surface/bone volume; Tb.Th, trabecular thickness; Tb.Sp, trabecular separation; Tb.N, trabecular number.
